# Poly[(μ_6_-naphthalene-1,4-dicarboxyl­ato-κ^6^ *O* ^1^:*O* ^1′^:*O* ^1′^:*O* ^4^:*O* ^4′^:*O* ^4′^)iron(II)]

**DOI:** 10.1107/S1600536808042669

**Published:** 2008-12-20

**Authors:** Jan Boeckmann, Inke Jess, Christian Näther

**Affiliations:** a Institut für Anorganische Chemie, Christian-Albrechts-Universität Kiel, Max-Eyth Strasse 2, D-24098 Kiel, Germany

## Abstract

In the title compound, [Fe(C_12_H_6_O_4_)]_*n*_, the Fe^II^ atom is coordinated by six O atoms from six symmetrically equivalent naphthalene-1,4-dicarboxyl­ate ligands in a strongly distorted octa­hedral geometry. These octa­hedra are connected *via* common edges into chains that elongate along the *a* axis, with Fe⋯Fe distances of 2.9712 (4) and 2.9724 (4) Å. The chains are linked *via* the naphthalene-1,4-dicarboxyl­ate ligands into a three-dimensional coordination network.

## Related literature

For isotypical structures with Mn^II^ and Co^II^, see: Maji *et al.* (2005 ▶).
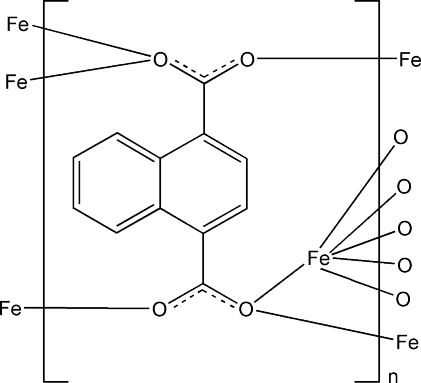


## Experimental

### 

#### Crystal data

[Fe(C_12_H_6_O_4_)]
*M* *_r_* = 270.02
Monoclinic, 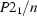

*a* = 4.7863 (4) Å
*b* = 14.8940 (9) Å
*c* = 13.4705 (10) Å
β = 91.098 (9)°
*V* = 960.10 (12) Å^3^
*Z* = 4
Mo *K*α radiation
μ = 1.57 mm^−1^
*T* = 170 (2) K
0.30 × 0.04 × 0.04 mm

#### Data collection

Stoe IPDS-1 diffractometer
Absorption correction: none
13722 measured reflections
2256 independent reflections
1816 reflections with *I* > 2σ(*I*)
*R*_int_ = 0.026

#### Refinement

*R*[*F* ^2^ > 2σ(*F* ^2^)] = 0.030
*wR*(*F* ^2^) = 0.087
*S* = 1.06
2256 reflections
155 parameters
H-atom parameters constrained
Δρ_max_ = 0.40 e Å^−3^
Δρ_min_ = −0.46 e Å^−3^

### 

Data collection: *IPDS* (Stoe & Cie, 1998 ▶); cell refinement: *IPDS*; data reduction: *IPDS*; program(s) used to solve structure: *SHELXS97* (Sheldrick, 2008 ▶); program(s) used to refine structure: *SHELXL97* (Sheldrick, 2008 ▶); molecular graphics: *SHELXTL* (Sheldrick, 2008 ▶) and *DIAMOND* (Brandenburg, 1999 ▶); software used to prepare material for publication: *SHELXTL*.

## Supplementary Material

Crystal structure: contains datablocks I, global. DOI: 10.1107/S1600536808042669/hy2174sup1.cif

Structure factors: contains datablocks I. DOI: 10.1107/S1600536808042669/hy2174Isup2.hkl

Additional supplementary materials:  crystallographic information; 3D view; checkCIF report

## Figures and Tables

**Table 1:** Selected bond lengths (Å)

Fe1—O3^i^	2.0557 (11)
Fe1—O2^ii^	2.0604 (11)
Fe1—O1	2.1533 (13)
Fe1—O4^iii^	2.1550 (13)
Fe1—O4^iv^	2.1867 (11)
Fe1—O1^v^	2.1908 (11)
Fe1—Fe1^vi^	2.9712 (4)
Fe1—Fe1^v^	2.9724 (4)

Symmetry codes: (i) 
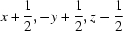
; (ii) 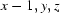; (iii) 
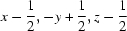
; (iv) 
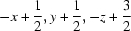
; (v) 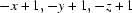; (vi) 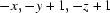.

**Fig. 1.**
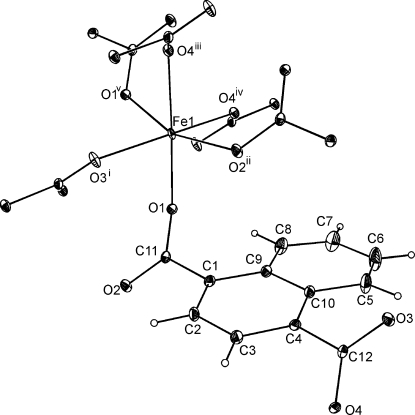
The structure of the title compound, together with symmetry-related atoms to complete the Fe coordination. Displacement ellipsoids are drawn at the 50% probability level. [Symmetry codes: (i) x+1/2, -y+1/2, z-1/2; (ii) x-1, y, z; (iii) x-1/2, -y+1/2, z-1/2; (iv) -x+1/2, y+1/2, -z+3/2; (v) -x+1, -y+1, -z+1.]

**Fig. 2.**
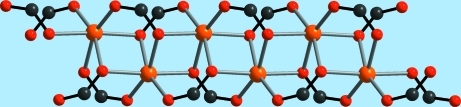
A view of the chains formed by the Fe coordination octahedra.

**Fig. 3.**
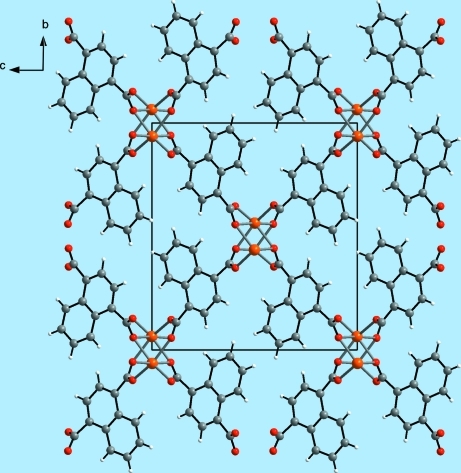
Three-dimensional structure of the title compound viewed along the a axis.

## supplementary crystallographic information

### Comment

The structure determination of the title compound was performed as a part of a project on the synthesis of new metal–organic frameworks. In this project we have reacted iron(II) sulfate with naphthalene-1,4-dicarboxylic acid in potassium hydroxide and water, which leads to the formation of a naphthalene-1,4-dicarboxylate iron(II) coordination polymer.

The title compound is isostructural to the manganese(II) and cobalt(II) complexes of naphthalene-1,4-dicarboxylate (Maji *et al.*, 2005). In the title compound, the Fe^II^ atom is surrounded by six O atoms from six crystallographically equivalent naphthalene-1,4-dicarboxylate ligands in a distorted octahedral coordination environment (Fig. 1 and Table 1). The Fe atoms are linked by O atoms of the carboxylate groups in a µ_3_-O:O':O' mode into chains, which elongate along the *a* axis (Fig. 2). Within these chains the Fe coordination octahedra are connected *via* common edges. These chains are connected by the naphthalene-1,4-dicarboxylate ligands into a three-dimensional network.

### Experimental

A mixture of FeSO_4_.7H_2_O (0.139 g, 0.5 mmol), naphthalene-1,4-dicarboxylic acid (0.108 g, 0.5 mmol), KOH (0.112 g, 1 mmol) and water (5 ml) was transfered into a glass tube and heated to 423 K for 4 d. On cooling, yellow needle crystals of the title compound were obtained.

### Refinement

H atoms were positioned geometrically and refined as riding atoms, with C—H = 0.95 Å and with *U*_iso_(H) = 1.2*U*_eq_(C).

### Crystal data

**Table:**

[Fe(C_12_H_6_O_4_)]	*F*(000) = 544
*M**_r_* = 270.02	*D*_x_ = 1.868 Mg m^−^^3^
Monoclinic, *P*2_1_/*n*	Mo *K*α radiation, λ = 0.71073 Å
Hall symbol: -P 2yn	Cell parameters from 8000 reflections
*a* = 4.7863 (4) Å	θ = 9–26°
*b* = 14.8940 (9) Å	µ = 1.57 mm^−^^1^
*c* = 13.4705 (10) Å	*T* = 170 K
β = 91.098 (9)°	Needle, yellow
*V* = 960.10 (12) Å^3^	0.30 × 0.04 × 0.04 mm
*Z* = 4	

### Data collection

**Table:**

Stoe IPDS-1 diffractometer	1816 reflections with *I* > 2σ(*I*)
Radiation source: fine-focus sealed tube	*R*_int_ = 0.026
graphite	θ_max_ = 28.0°, θ_min_ = 2.7°
φ scans	*h* = −6→6
13722 measured reflections	*k* = −19→19
2256 independent reflections	*l* = −17→17

### Refinement

**Table:**

Refinement on *F*^2^	Secondary atom site location: difference Fourier map
Least-squares matrix: full	Hydrogen site location: inferred from neighbouring sites
*R*[*F*^2^ > 2σ(*F*^2^)] = 0.030	H-atom parameters constrained
*wR*(*F*^2^) = 0.087	*w* = 1/[σ^2^(*F*_o_^2^) + (0.0535*P*)^2^ + 0.4222*P*] where *P* = (*F*_o_^2^ + 2*F*_c_^2^)/3
*S* = 1.06	(Δ/σ)_max_ = 0.002
2256 reflections	Δρ_max_ = 0.40 e Å^−^^3^
155 parameters	Δρ_min_ = −0.46 e Å^−^^3^
0 restraints	Extinction correction: *SHELXL97* (Sheldrick, 2008), Fc^*^=kFc[1+0.001xFc^2^λ^3^/sin(2θ)]^-1/4^
Primary atom site location: structure-invariant direct methods	Extinction coefficient: 0.012 (3)

### Fractional atomic coordinates and isotropic or equivalent isotropic displacement parameters (Å^2^)

**Table:**

	*x*	*y*	*z*	*U*_iso_*/*U*_eq_	
Fe1	0.24998 (4)	0.440859 (14)	0.501167 (14)	0.00529 (13)	
O1	0.6268 (3)	0.44515 (7)	0.59098 (9)	0.0071 (2)	
O2	1.0178 (2)	0.36165 (8)	0.59405 (9)	0.0095 (2)	
O3	−0.0120 (2)	0.13335 (8)	0.90581 (9)	0.0099 (2)	
O4	0.3838 (3)	0.05263 (7)	0.90583 (9)	0.0067 (2)	
C1	0.6451 (3)	0.31639 (10)	0.69582 (11)	0.0077 (3)	
C2	0.6838 (4)	0.22537 (11)	0.68337 (12)	0.0097 (3)	
H2	0.8026	0.2044	0.6326	0.012*	
C3	0.5492 (4)	0.16326 (11)	0.74501 (12)	0.0097 (3)	
H3	0.5782	0.1008	0.7353	0.012*	
C4	0.3761 (3)	0.19163 (10)	0.81920 (11)	0.0074 (3)	
C5	0.1863 (4)	0.31859 (12)	0.91907 (13)	0.0165 (4)	
H5	0.0869	0.2776	0.9593	0.020*	
C6	0.1788 (5)	0.40864 (12)	0.94029 (14)	0.0233 (5)	
H6	0.0777	0.4292	0.9959	0.028*	
C7	0.3198 (5)	0.47108 (12)	0.88038 (13)	0.0199 (4)	
H7	0.3155	0.5332	0.8963	0.024*	
C8	0.4628 (4)	0.44215 (10)	0.79915 (13)	0.0135 (4)	
H8	0.5519	0.4848	0.7579	0.016*	
C9	0.4798 (3)	0.34898 (10)	0.77570 (11)	0.0081 (3)	
C10	0.3413 (3)	0.28589 (11)	0.83760 (11)	0.0084 (3)	
C11	0.7751 (3)	0.37880 (10)	0.62232 (11)	0.0067 (3)	
C12	0.2371 (3)	0.12168 (10)	0.88160 (11)	0.0067 (3)	

### Atomic displacement parameters (Å^2^)

**Table:**

	*U*^11^	*U*^22^	*U*^33^	*U*^12^	*U*^13^	*U*^23^
Fe1	0.00470 (18)	0.00449 (16)	0.00679 (17)	0.00009 (8)	0.00311 (10)	0.00003 (7)
O1	0.0072 (6)	0.0065 (5)	0.0076 (5)	0.0000 (4)	0.0012 (4)	0.0020 (4)
O2	0.0071 (6)	0.0088 (5)	0.0129 (5)	0.0000 (4)	0.0048 (4)	0.0036 (4)
O3	0.0063 (6)	0.0095 (5)	0.0139 (5)	−0.0002 (4)	0.0036 (4)	0.0044 (4)
O4	0.0071 (6)	0.0056 (5)	0.0074 (5)	−0.0002 (4)	0.0009 (4)	0.0023 (4)
C1	0.0069 (7)	0.0085 (7)	0.0078 (7)	−0.0012 (6)	0.0011 (6)	0.0027 (5)
C2	0.0103 (8)	0.0096 (7)	0.0094 (7)	0.0010 (6)	0.0048 (6)	0.0017 (5)
C3	0.0116 (8)	0.0071 (7)	0.0104 (7)	0.0008 (5)	0.0032 (6)	0.0020 (6)
C4	0.0068 (8)	0.0071 (7)	0.0082 (6)	−0.0004 (5)	0.0009 (5)	0.0029 (5)
C5	0.0230 (10)	0.0120 (8)	0.0150 (8)	0.0004 (7)	0.0117 (7)	0.0020 (6)
C6	0.0367 (12)	0.0128 (9)	0.0210 (9)	0.0038 (8)	0.0183 (8)	−0.0011 (7)
C7	0.0338 (11)	0.0089 (8)	0.0173 (8)	0.0011 (7)	0.0100 (8)	−0.0005 (6)
C8	0.0207 (10)	0.0077 (8)	0.0122 (7)	−0.0020 (6)	0.0050 (7)	0.0011 (6)
C9	0.0084 (8)	0.0075 (7)	0.0083 (7)	−0.0003 (5)	0.0017 (6)	0.0017 (5)
C10	0.0091 (8)	0.0081 (7)	0.0080 (7)	0.0004 (5)	0.0025 (6)	0.0016 (5)
C11	0.0074 (8)	0.0066 (7)	0.0061 (6)	−0.0018 (5)	0.0012 (5)	0.0000 (5)
C12	0.0076 (8)	0.0070 (7)	0.0056 (6)	−0.0011 (5)	0.0015 (5)	0.0004 (5)

### Geometric parameters (Å, °)

**Table:**

Fe1—O3^i^	2.0557 (11)	C2—H2	0.9500
Fe1—O2^ii^	2.0604 (11)	C3—C4	1.377 (2)
Fe1—O1	2.1533 (13)	C3—H3	0.9500
Fe1—O4^iii^	2.1550 (13)	C4—C10	1.436 (2)
Fe1—O4^iv^	2.1867 (11)	C4—C12	1.502 (2)
Fe1—O1^v^	2.1908 (11)	C5—C6	1.372 (3)
Fe1—Fe1^vi^	2.9712 (4)	C5—C10	1.422 (2)
Fe1—Fe1^v^	2.9724 (4)	C5—H5	0.9500
O1—C11	1.2835 (19)	C6—C7	1.411 (3)
O2—C11	1.256 (2)	C6—H6	0.9500
O3—C12	1.254 (2)	C7—C8	1.371 (2)
O4—C12	1.2839 (19)	C7—H7	0.9500
C1—C2	1.379 (2)	C8—C9	1.426 (2)
C1—C9	1.433 (2)	C8—H8	0.9500
C1—C11	1.502 (2)	C9—C10	1.428 (2)
C2—C3	1.407 (2)		
			
O3^i^—Fe1—O2^ii^	112.54 (5)	C2—C1—C9	120.12 (14)
O3^i^—Fe1—O1	84.19 (5)	C2—C1—C11	118.04 (13)
O2^ii^—Fe1—O1	97.55 (5)	C9—C1—C11	121.82 (14)
O3^i^—Fe1—O4^iii^	96.08 (5)	C1—C2—C3	120.69 (15)
O2^ii^—Fe1—O4^iii^	86.89 (5)	C1—C2—H2	119.7
O1—Fe1—O4^iii^	175.08 (4)	C3—C2—H2	119.7
O3^i^—Fe1—O4^iv^	159.92 (5)	C4—C3—C2	121.01 (15)
O2^ii^—Fe1—O4^iv^	85.45 (4)	C4—C3—H3	119.5
O1—Fe1—O4^iv^	84.65 (4)	C2—C3—H3	119.5
O4^iii^—Fe1—O4^iv^	93.64 (4)	C3—C4—C10	119.93 (14)
O3^i^—Fe1—O1^v^	84.50 (4)	C3—C4—C12	118.20 (14)
O2^ii^—Fe1—O1^v^	160.39 (5)	C10—C4—C12	121.83 (13)
O1—Fe1—O1^v^	93.65 (4)	C6—C5—C10	120.77 (16)
O4^iii^—Fe1—O1^v^	81.50 (4)	C6—C5—H5	119.6
O4^iv^—Fe1—O1^v^	79.61 (5)	C10—C5—H5	119.6
O3^i^—Fe1—Fe1^vi^	140.19 (4)	C5—C6—C7	120.69 (16)
O2^ii^—Fe1—Fe1^vi^	84.39 (3)	C5—C6—H6	119.7
O1—Fe1—Fe1^vi^	130.85 (3)	C7—C6—H6	119.7
O4^iii^—Fe1—Fe1^vi^	47.26 (3)	C8—C7—C6	120.04 (16)
O4^iv^—Fe1—Fe1^vi^	46.37 (3)	C8—C7—H7	120.0
O1^v^—Fe1—Fe1^vi^	76.12 (3)	C6—C7—H7	120.0
O3^i^—Fe1—Fe1^v^	81.72 (3)	C7—C8—C9	120.93 (15)
O2^ii^—Fe1—Fe1^v^	142.14 (4)	C7—C8—H8	119.5
O1—Fe1—Fe1^v^	47.35 (3)	C9—C8—H8	119.5
O4^iii^—Fe1—Fe1^v^	127.79 (3)	C8—C9—C10	118.82 (14)
O4^iv^—Fe1—Fe1^v^	78.44 (3)	C8—C9—C1	122.06 (14)
O1^v^—Fe1—Fe1^v^	46.30 (3)	C10—C9—C1	119.01 (14)
Fe1^vi^—Fe1—Fe1^v^	107.275 (14)	C5—C10—C9	118.68 (14)
C11—O1—Fe1	127.88 (10)	C5—C10—C4	122.14 (14)
C11—O1—Fe1^v^	129.03 (11)	C9—C10—C4	119.07 (13)
Fe1—O1—Fe1^v^	86.35 (4)	O2—C11—O1	124.47 (14)
C11—O2—Fe1^vii^	125.49 (10)	O2—C11—C1	118.13 (14)
C12—O3—Fe1^viii^	128.98 (10)	O1—C11—C1	117.38 (14)
C12—O4—Fe1^ix^	123.36 (10)	O3—C12—O4	124.25 (14)
C12—O4—Fe1^x^	126.21 (10)	O3—C12—C4	118.88 (14)
Fe1^ix^—O4—Fe1^x^	86.36 (4)	O4—C12—C4	116.87 (14)

Symmetry codes: (i) *x*+1/2, −*y*+1/2, *z*−1/2; (ii) *x*−1, *y*, *z*; (iii) *x*−1/2, −*y*+1/2, *z*−1/2; (iv) −*x*+1/2, *y*+1/2, −*z*+3/2; (v) −*x*+1, −*y*+1, −*z*+1; (vi) −*x*, −*y*+1, −*z*+1; (vii) *x*+1, *y*, *z*; (viii) *x*−1/2, −*y*+1/2, *z*+1/2; (ix) *x*+1/2, −*y*+1/2, *z*+1/2; (x) −*x*+1/2, *y*−1/2, −*z*+3/2.
